# ﻿Description of two new species of *Conostigmus* Dahlbom, 1858 (Hymenoptera, Megaspilidae) from Yintiaoling Nature Reserve, Chongqing, China

**DOI:** 10.3897/zookeys.1255.154710

**Published:** 2025-10-15

**Authors:** Yuanhan Yang, Fang Li, Yixin Huang, Xu Wang, Zhisheng Zhang, Huayan Chen

**Affiliations:** 1 Collaborative Innovation Center of Recovery and Reconstruction of Degraded Ecosystem in Wanjiang Basin Co-founded by Anhui Province and Ministry of Education; School of Ecology and Environment, Anhui Normal University, Wuhu, Anhui 241000, China; 2 Anhui Provincial Key Laboratory of the Conservation and Exploitation of Biological Resources, Key Laboratory of Biotic Environment and Ecological Safety in Anhui Province, College of Life Sciences, Anhui Normal University, Wuhu, Anhui 241000, China; 3 Key Laboratory of Eco-environments in Three Gorges Reservoir Region (Ministry of Education), School of Life Sciences, Southwest University, Chongqing 400715, China; 4 Guangdong Provincial Key Laboratory of Applied Botany, South China Botanical Garden, Chinese Academy of Sciences, Guangzhou 510650, China

**Keywords:** 28S rDNA, Ceraphronoidea, identification key, morphology, parasitoid wasps, taxonomy

## Abstract

Two new species of *Conostigmus* Dahlbom, 1858, *C. rotundus* Yang & Wang, **sp. nov.** and *C. clayulatus* Yang & Wang, **sp. nov.** are described from the Yintiaoling Nature Reserve, Chongqing, China. The 28S rDNA sequences of the new species were generated to associate the female and male of the same species.

## ﻿Introduction

Megaspilidae belongs to Ceraphronoidea of Hymenoptera. It is widely distributed throughout the world except for the polar regions (Johnson and Musetti 2004; Mikó et al. 2011; Bijoy et al. 2014; Pezzini and Köhler 2020). More than 450 species of 12 genera are known, and recent studies indicate that a very high number of undescribed species exist in Megaspilidae (Iemma et al. 2016; Salden and Peters 2023). *Conostigmus* was established by Dahlbom (1858) and proposed as a subgenus of *Megaspilus*. Kieffer (1907) erected *Conostigmus* as a genus from *Megaspilus*. *Conostigmus* is the most species-rich and widely distributed group in the family Megaspilidae, with about 180 species recorded worldwide (Johnson and Musetti 2004; Trietsch et al. 2020; Cui et al. 2023; Qian et al. 2024; Wang et al. 2024).

Considering that sexual dimorphism is common in *Conostigmus*, it is difficult to match male and female individuals of the same species by morphological identification alone. Molecular identification is necessary to associate both sexes of the same species.

In the present paper, we describe two new species of *Conostigmus*, *C. rotundus* Yang & Wang, sp. nov., and *C. clayulatus* Yang & Wang, sp. nov. from Chongqing, China, bringing the Chinese species number of *Conostigmus* up to ten (Dessart 1997; Cui et al. 2023; Qian et al. 2024; Wang et al. 2024). In addition, we provide 28S rDNA sequences of the two new species, which contribute to the molecular systematics of *Conostigmus*.

## ﻿Material and methods

The specimens in this study were collected from sweep-net and yellow pan traps. Specimens were deposited in the Insect Collection of Anhui Normal University (AHNU), Wuhu, China.

All voucher specimens were then observed and dried in the air. The specimens were photographed using a Leica M205A stereo microscope and a Leica DFC-500 digital camera. Image stacking was performed with Leica Microsystems CMS GmbH. To facilitate the preparation of male genitalia for morphological study, apical metasomal segments were dissected from specimens and subjected to a graded chemical processing protocol: first incubated in 35% H_2_O_2_ for 24 h, then transferred to 5% acetic acid for 24 h of further treatment, followed by a 1-h rinse in distilled water. After post-processing, the segments were transferred to a glycerin microdroplet on a concavity slide, and subsequent microdissection was performed in glycerin using #5 fine forceps and #2 insect pins as dissecting instruments. Measurements are given in microns. Morphological terminology follows the Hymenoptera Anatomy Ontology (Yoder et al. 2010) (Table 1).

**Table 1.** Abbreviations and morphological terms used in text.

F1, F2, ..., F9	Flagellum 1, 2, ..., F9.
LOL	Lateral ocellar length, shortest distance between inner margins of median and lateral ocelli.
OOL	Ocular ocellar length, minimum distance between a posterior ocellus to the eye margin.
POL	Posterior ocellar length, shortest distance between inner margins of posterior ocelli.
HH	Head height, lateral.
EHf	Eye height, anterior view.
HL	Head length.
HW	Head width.
IOS	Interorbital space.
AseW	Anterior mesoscutal width.
PscW	Posterior mesoscutal width.

According to previous studies, sexual dimorphism is prevalent in *Conostigmus* (Trietsch et al. 2020; Wang et al. 2024). In order to match females and males of the same species, we sequenced the 28S rDNA of each species. A total of eight male and female sequences were generated for five species, including the two new species (two of which were published only for males). DNA was extracted from each putative species using the TIANamp Genomic DNA Kit (TIANGEN, Changping District, Beijing, cat. no. DP3400), following the protocol used by Taekul et al. (2014). Primers used for 28S amplification were D2-3549 (5’-AGTCGTGTTGCTTGATAGTGCAG-3’) and D2-4068 (5’-TTGGTCCGTGTTTCAAGACGGG-3’) (Zhang et al. 2014). The polymerase chain reactions (PCRs) were performed in a 25 μL reaction volume in a T100 Thermal Circulator (Bio-Rad). The thermal cycle conditions were: an initial denaturation step of 94 °C for 1 min, followed by 35 cycles of 94 °C for 1 min, 50 °C for 30 s, 72 °C for 30 s, with an additional extension at 72 °C for 5 min. Amplicons were directly sequenced in both directions with forward and reverse primers by GENERAL BIOL (Anhui, China). Chromatograms were assembled with Sequencing Analysis 6 (Thermo Fisher Scientific, Gloucester, UK). Sequences generated from this study were deposited in GenBank (accession numbers are listed in Table 2). *Conostigmus* sequences from the previous studies were downloaded from GenBank (Table 2).

**Table 2.** Detailed information of sequenced samples and accession numbers.

Species	Sex	GenBank accession No.	Reference
*D. carpenteri*	male	MZ340592	Wang et al. (2021)
	female	MZ340590	Wang et al. (2021)
*D. laticeps*	male	MZ340593	Wang et al. (2021)
	female	MZ340624	Wang et al. (2021)
*D. bellus*	male	MZ344975	Wang et al. (2021)
	female	MZ344976	Wang et al. (2021)
*D. anisodontus*	male	MZ344977	Wang et al. (2021)
	female	MZ344978	Wang et al. (2021)
*D. lui*	male	OR120392	Li et al. (2023)
	female	OR120391	Li et al. (2023)
*C. xui*	male	SAMN44283636	this study
*C. nankunensis*	male	SAMN44283637	this study
*C. ampullaceus*	male	SAMN44283638	this study
	female	SAMN44283639	this study
*C. rotundus* sp. nov.	male	SAMN44283640	this study
	female	SAMN44283641	this study
*C. clayulatus* sp. nov.	male	SAMN44283642	this study
	female	PQ686133	this study

The genetic distances were calculated under the Kimura 2-parameter (K2P) model in MEGA X (Kumar et al. 2018). The sequences were aligned using the MUSCLE (Multiple Sequence Comparison by Log-Expectation) model of MEGA X (Pais et al. 2014). The alignment was used to reconstruct a maximum-likelihood (ML) tree by using the Auto-detect model of IQ-TREE (Minh et al. 2020). The specific parameters were set to Ultrafast for Bootstrap analysis with 1000 bootstrap alignments and 1000 maximum iterations. The minimum correlation coefficient was set to 0.99, and the SH-aLRT branch test replicates were set to 1000. The ingroup consists of five *Conostigmus* and five *Dendrocerus* species. Sequences of two *Ceraphron* species (accession Nos. MH733890 and GQ374733) were downloaded from GenBank and used as outgroups.

## ﻿Results

### ﻿Taxonomy

#### ﻿ Conostigmus

Taxon classificationAnimaliaHymenopteraMegaspilidae

Dahlbom, 1858

E36703DD-3C83-5CA3-A6F8-D171491E4E4F

Conostigmus Dahlbom, 1858: 291.
Dichogmus Thomson, 1858: 287.
Eumegaspilus Ashmead, 1888: 48.
Eumegalospilus : Schulz, 1906: 152.
Conostigmoides Dodd, 1914: 88.
Ecnomothorax Dessart & Masner, 1965: 276.
Dolichoceraphron Hellén, 1966: 15.
Szelenyides Dessart, 1974: 43.

### ﻿Key to the species of *Conostigmus* from China (male)

**Table:** 

1	Mesosoma distinctly elongate, length more than 2.0 × maximum width	***C. ampullaceus* Dessert, 1997**
–	Mesosoma moderately narrow, length 2.0 × or less than maximum width	**2**
2	Facial pit absent	**3**
–	Facial pit present	**6**
3	Anteromedian projection of metanoto-propodeo-metapecto-mesopectal complex present	**4**
–	Anteromedian projection of metanoto-propodeo-metapecto-mesopectal complex absent	**5**
4	Sternaulus extending to 2/3 length of mesopleuron	***C. asperatus* Wang & Zhu, 2025**
–	Sternaulus extending to 4/5 length of mesopleuron; facial sulcus present	***C. clayulatus* Yang & Wang, sp. nov.**
5	Harpe shorter than gonostipes	***C. nankunensis* Qian & Wang, 2024**
–	Harpe longer than gonostipes	***C. abdominalis* Boheman, 1832**
6	Flagellomere 1 (F1) longer than scape	***C. longus* Wang & Zhu, 2025**
–	Flagellomere 1 (F1) shorter than or equal in length to scape	**7**
7	Body length (excluding antennae) ≥ 2.0 mm	**8**
–	Body length (excluding antennae) < 2.0 mm	**10**
8	Basal gastral carinae length ≥ 1/3 of syntergum length	**9**
–	Basal gastral carinae length < 1/3 of syntergum length; harpe longer than gonostipes	***C. xui* Cui & Wang, 2023**
9	F1 subequal to scape; head and mesosoma reddish brown	***C. electrinus* Wang & Chen, 2024**
–	F1 shorter than scape; head and mesosoma black	***C. villosus* Dessert, 1997**
10	Preoccipital furrow present and extends into the ocellar triangle	***C. acutus* Wang & Chen, 2024**
–	Preoccipital furrow present but does not extend into the ocellar triangle	**11**
11	Scutellum as long as wide; dorsal margin of S9 straight	***C. rotundus* Yang & Wang, sp. nov.**
–	Scutellum longer than wide; dorsal margin of S9 protruded	***C. quadripetalus* Wang & Chen, 2024**

#### ﻿ Conostigmus rotundus

Taxon classificationAnimaliaHymenopteraMegaspilidae

Yang & Wang sp. nov.

1E820167-69CD-5469-9D5A-78B40E3CF239

https://zoobank.org/C2EF15F6-E88E-4800-A927-D6CD28221BBD

##### Diagnosis.

The new species can be distinguished from other *Conostigmus* species by the following characters: facial sulcus absent; sternaulus present, elongate and reaching 4/5 of mesopleuron length; scutellum as long as wide; harpe simple, not bilobed, distal margin of harpe straight, and pointed laterally; and S9 bowl shaped, concave tip.

##### Material examined.

***Holotype*.** ♂, China • Chongqing Municipality, Wuxi County, Yintiaoling Nature Reserve, Mt. Guanshan, 31°31'5.65"N, 109°42'59.07"E, alt. 1978 m, 22 July 2024, F. Li, W. J. Zhao, Y. C. Li leg. (CQWX-2402-0701-29). ***Paratypes*.** 1♂1♀, China • Chongqing Municipality, Wuxi County, Yintiaoling Nature Reserve, Mt. Guanshan, 31°31'5.65"N, 109°42'59.07"E, alt. 1978 m, 22 July 2024, F. Li, W. J. Zhao, Y. C. Li leg. (CQWX-2402-0701-28/32).

##### Description.

**Male**: Body length 1.4–1.6 mm (*N* = 2).

**Figure 1.**
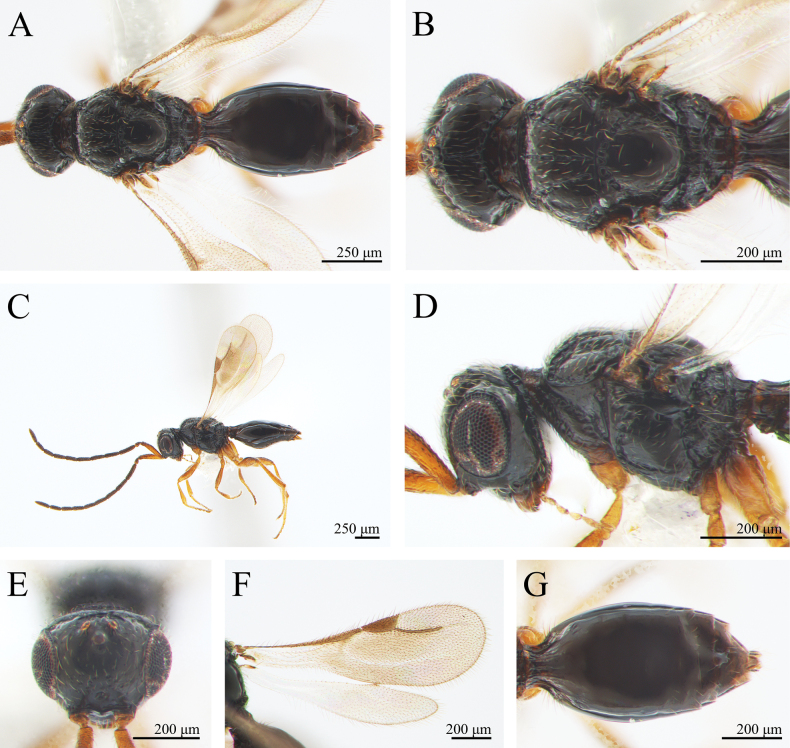
*Conostigmus rotundus* Yang & Wang, sp. nov., male, holotype. A. Dorsal habitus; B. Head and mesosoma, dorsal view; C. Lateral habitus; D. Head and mesosoma, lateral view; E. Head, anterior view; F. Wings; G. Metasoma, dorsal view.

***Coloration*** (Fig. 1). Cranium, mesosoma and metasoma black. Mandibles brown palps yellow. Scape and pedicel brown, F1 to F9 black. Coxa of fore, mid and hind legs black to brown; rest of legs brownish yellow. Syntergum black; posterior metasomal segments black (Fig. 1G). Pterostigma, costal vein, and radial vein light brown (Fig. 1F). Body pubescence pale yellowish brown; marginal fringes of wings light brown.

***Head*** (Fig. 1B, D, E). About the width of mesosoma (about 1.02 × as wide as mesosoma). HH: EHf = 1.7–1.9. HH: HL = 1.6–1.7. HW: IOS = 1.4–1.6. HW: HH = 1.1. OOL longer than POL and ocellar triangle with narrow base. OOL: LOL = 2.9–3.0. POL: OOL = 0.6–0.7. Head oval with pubescence. Preoccipital lunula present. Preoccipital furrow present, ending inside ocellar triangle, but posterior to anterior ocellus. Median process on intertorular carina present, process not extending across intertorular area towards dorsal margin of clypeus. Facial sulcus absent. Facial pit present. Intertorular carina present.

***Antennae*** (Fig. 1C). Scape 1.1 × as long as the combined length of pedicel and F1. Scape length vs. pedicel length: 3.5–3.6. Scape length vs. F1 length: 1.5. F1 length vs. pedicel length: 2.5–2.6. F1 length vs. F2 length: 1.1. Flagellum cylindrical. Setae on flagellomeres shorter than the width of flagellomeres.

***Mesosoma*** (Fig. 1B, D). Pronotum shorter than the mesoscutum along the midline. AscW/PscW = 1.1. Mesosoma 1.3 × as long as wide (Length/width/height = 526/396/402 μm), densely pubescent. Mesoscutum 2.0 × as wide as long (Width/length = 397/195 μm). Transscutal articulation present. Median mesoscutal sulcus present. Notaulus present, foveate and extends the length of the mesoscutum (percurrent), not adjacent to the median mesoscutal sulcus posteriorly. Scutoscutellar sulcus present and foveolate. Scutoscutellar sulcus adjacent and contiguous to transscutal articulation. Axillular carinae present. Scutellum as long as wide. Sternaulus present, elongate and reaching 4/5 of mesopleuron length. Anterior mesopleural sulcus present. Mesopleural sulcus shape: straight. Mesopleural pit present. Lateral propodeal carina forming an inverted “Y” shape.

***Wing*** (Fig. 1F). Fore wing total length 1.2–1.3 mm, translucent with nearly triangular pterostigma. Pterostigma length vs. width: 3.1. Radius (242 μm), slightly curved medially, 1.2 × as long as the length of the pterostigma. Fore wing with translucent orange-brown irregular stripes and dense light brown pubescence; wing marginal hairs longer than those on the inner wing surface; hind wing venation reduced, translucent.

***Metasoma*** (Fig. 1G). Metasoma 1.9 × as long as wide (Length/width/height = 768/413/322 μm). Metasoma shape: ovoid. Syntergum with five distinct gastral carinae, reaching 1/3 of syntergum length; syntergal translucent patch transverse. Rest of tergites smooth, with sparse hairs on both sides.

**Figure 2.**
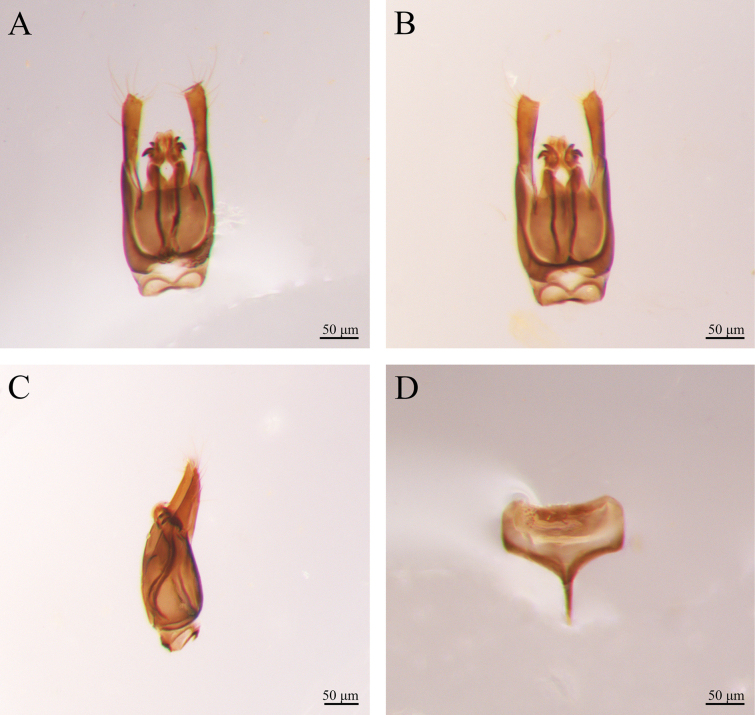
*Conostigmus rotundus* Yang & Wang, sp. nov., male, holotype, genitalia. A. Dorsal view; B. Ventral view; C. Lateral view D S9.

***Male genitalia*** (Fig. 2). Harpe slightly longer than gonostipes, with numerous slender apical setae; harpe orientation: medial; harpe shape: simple and not bilobed, distal margin straight dorsally and ventrally, and pointed; lateral setae of harpe present, but sparse. Parossiculus not fused medially. Gonostipes longer than wide, not fused with parossiculus. Penisvalva curved ventrally. Gonossiculus and gonossiculus spine present; apical parossiculal setae present. The middle part of male S9 slightly concave. The spiculum of S9 short, reaching 1/3 of S9.

**Figure 3.**
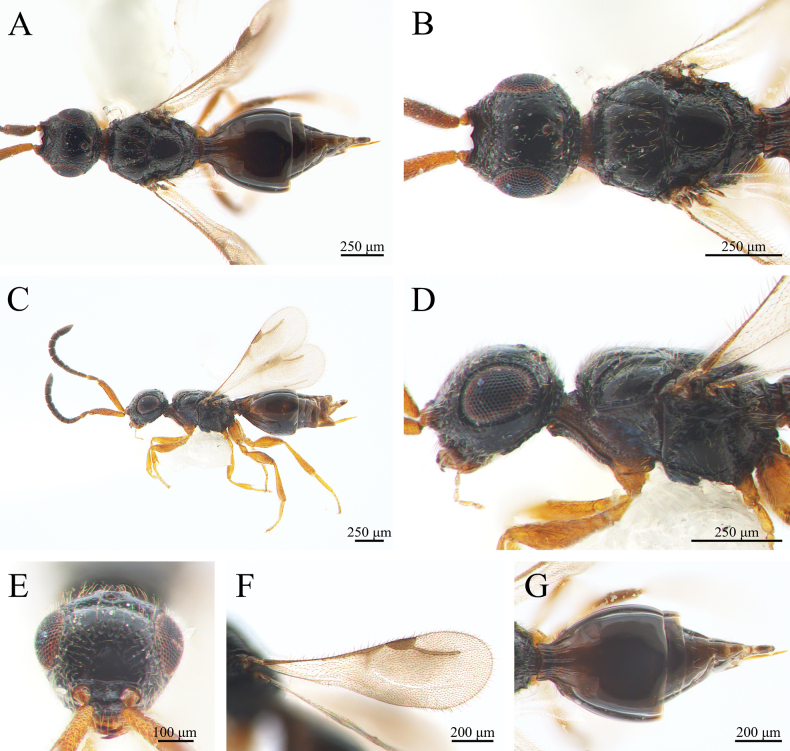
*Conostigmus rotundus* Yang & Wang, sp. nov., female, holotype. A. Dorsal habitus; B. Head and mesosoma, dorsal view; C. Lateral habitus; D. Head and mesosoma, lateral view; E. Head, anterior view; F. Wings; G. Metasoma, dorsal view.

**Female** (Fig. 3). Same as the males, except for the following characters: body length = 1.9 mm; antennal pedicel long, slightly longer than any individual flagellomere from F1 to F8 of the same individual; legs tawny, coxa blackish-brown; wider mesosoma (Length/width = 554/451 μm).

##### Remarks.

This new species is similar to *C. acutus* Wang & Cui, 2024 in body length, preoccipital lunula, preoccipital furrow and lateral propodeal carina but can be distinguished by the almost circular scutellum (subcircular, subequal in length and width, more distinct than that in *C. acutus*); and the harpe straight apically and distal margin pointed in lateral view (harpe outward apically and distal margin beveled in lateral view). And this new species is similar to *C. quadripetalus* Wang & Chen, 2024 in the preoccipital lunula, preoccipital furrow, syntergum and harpe shape, but can be distinguished by body length (a longer body length than *C. quadripetalus*); S9 shape (male S9 shape of *C. quadripetalus* convex in distal margin but concave in *C. rotundus*); and HH: HL (a higher head height than *C. quadripetalus*).

##### Distribution.

China (Chongqing).

##### Etymology.

Consistent with the genus name, the species name is a Latin masculine adjective meaning “rounded”, referring to the nearly round scutellum of this species.

#### ﻿ Conostigmus clayulatus

Taxon classificationAnimaliaHymenopteraMegaspilidae

Yang & Wang sp. nov.

B1835746-2686-5328-9F06-357CAA0FBA51

https://zoobank.org/11E831F1-6770-49DE-AEC3-144A21A7BB05

##### Diagnosis.

The new species can be distinguished from other *Conostigmus* species by the following characters: facial sulcus present and extends from the median ocellus to the intertorular carina; sternaulus present, elongate and reaching 3/4 of mesopleuron length; harpe with numerous long and slender apical setae and sparse lateral setae; and S9 cup-shaped with a straight tip bearing six setal patches at the end.

##### Material examined.

***Holotype*.** ♂, China • Chongqing Municipality, Wuxi County, Yintiaoling Nature Reserve, Cotton Hill, 31°30'38.35"N, 109°42'4.54"E, alt. 2188 m, 22 July 2024, F. Li, W. J. Zhao, Y. C. Li leg. (CQWX-2402-0707-18). ***Paratypes*.** 1♂, China • Chongqing Municipality, Wuxi County, Yintiaoling Nature Reserve, Mt. Guanshan, 31°32'12.43"N, 109°41'58.38"E, alt. 2155 m, 21 July 2024, F. Li, W. J. Zhao, Y. C. Li leg. (CQWX-2402-0702-13). 1♀, China • Chongqing Municipality, Wuxi County, Yintiaoling Nature Reserve, Cotton Hill, 31°30'38.35"N, 109°42'4.54"E, alt. 2188 m, 22 July 2024, F. Li, W. J. Zhao, Y. C. Li leg. (CQWX-2402-0707-20).

##### Description.

**Male**: Body length 2.1–2.2 mm (*N* = 2).

**Figure 4.**
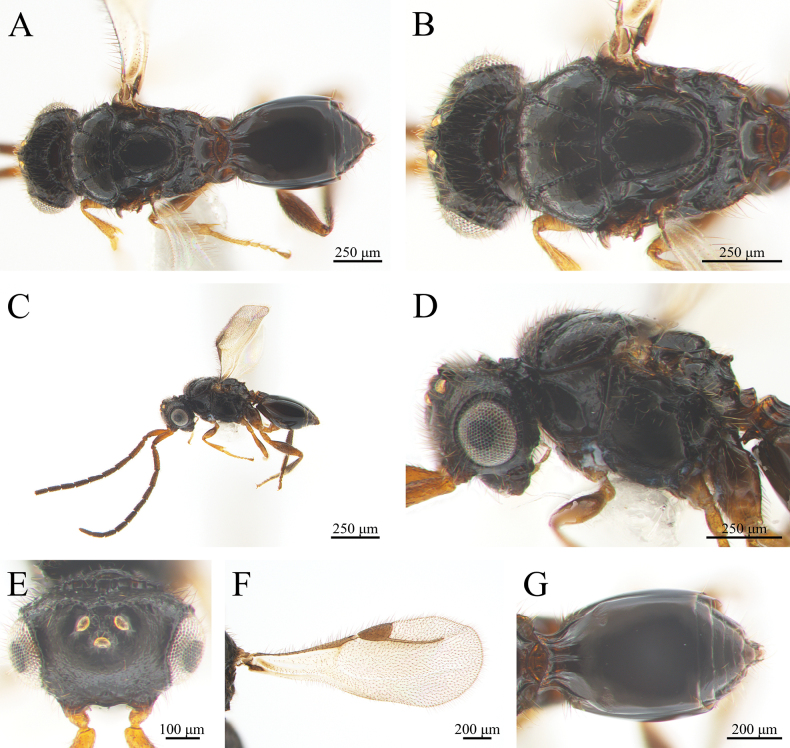
*Conostigmus clayulatus* Yang & Wang, sp. nov. male, holotype. A. Dorsal habitus; B. Head and mesosoma, dorsal view; C. Lateral habitus; D. Head and mesosoma, lateral view; E. Head, anterior view; F. Wings; G. Metasoma, dorsal view.

***Coloration*** (Fig. 4). Cranium, mesosoma and metasoma black. Mandibles brown and palps yellowish brown. Scape, pedicel and F1 brown, F2 to F9 black. Legs and joint brownish yellow. Syntergum black (Fig. 4G); metasoma gaster black. Pterostigma, costal vein, and radial vein brown (Fig. 4F). Body pubescence pale yellowish brown; marginal fringes of wings light brown.

***Head*** (Fig. 4B, D, E). About the width of mesosoma. (about 0.96 × as wide as mesosoma). HH: EHf = 1.5–1.7. HH: HL = 0.7–1.1. HW: IOS = 1.4. HW: HH = 1.5–1.6. OOL longer than POL and ocellar triangle with narrow base. OOL: LOL = 2.6–2.9. POL: OOL = 0.6. Head oval with pubescence. Preoccipital lunula present. Preoccipital furrow present, ending inside ocellar triangle, but posterior to anterior ocellus. Median process on intertorular carina present, process not extending across intertorular area towards dorsal margin of clypeus. Facial sulcus present and extends from the median ocellus to the intertorular carina. Facial pit absent. Intertorular carina present.

***Antennae*** (Fig. 4C). Scape 0.7 × as long as the combined length of pedicel and F1. Scape length vs. pedicel length: 3.5–3.6. Scape length vs. F1 length: 1.0. F1 length vs. pedicel length: 3.5–3.7. F1 length vs. F2 length: 1.2–1.3. Flagellum cylindrical. Setae on flagellomeres shorter than the width of flagellomeres.

***Mesosoma*** (Fig. 4B, D). Pronotum shorter than the mesoscutum along the midline. AscW/PscW = 1.0. Mesosoma 1.6 × as long as wide (Length/width/height = 863/547/530 μm), densely pubescent. Mesoscutum 2.1 × as wide as long (Width/length = 547/264 μm). Transscutal articulation present. Median mesoscutal sulcus present. Notaulus present, straight and extends the length of the mesoscutum (percurrent), not connected to the median mesoscutal sulcus posteriorly. Scutoscutellar sulcus present and foveolate. Scutoscutellar sulcus adjacent and contiguous to transscutal articulation. Mesoscutellum 1.1 × as long as wide. Axillular carinae absent. Sternaulus present, elongate and reaching 3/4 of mesopleuron length. Anterior mesopleural sulcus present. Mesopleural sulcus shape: straight. Mesopleural pit present. Lateral propodeal carina forming an inverted “Y” shape.

***Wing*** (Fig. 4F). Fore wing total length 1.7 mm, translucent with relatively nearly triangular pterostigma. Pterostigma length vs. width: 2.1–2.3. Radius (408 μm), slightly curved medially, 1.6 × as long as the length of pterostigma. Fore wing with translucent brown irregular stripes and dense orange-brown pubescence; wing marginal hairs longer than those on the inner wing surface; hind wing venation reduced, translucent.

***Metasoma*** (Fig. 4G). Metasoma 1.7 × as long as wide (Length/width/height = 814/487/390 μm). Metasoma shape: olive-shaped or oval. Syntergum with five distinct gastral carinae, reaching 1/5 of syntergum length; syntergal translucent patch transverse. Rest of tergites smooth, but with sparse hairs on both sides.

**Figure 5.**
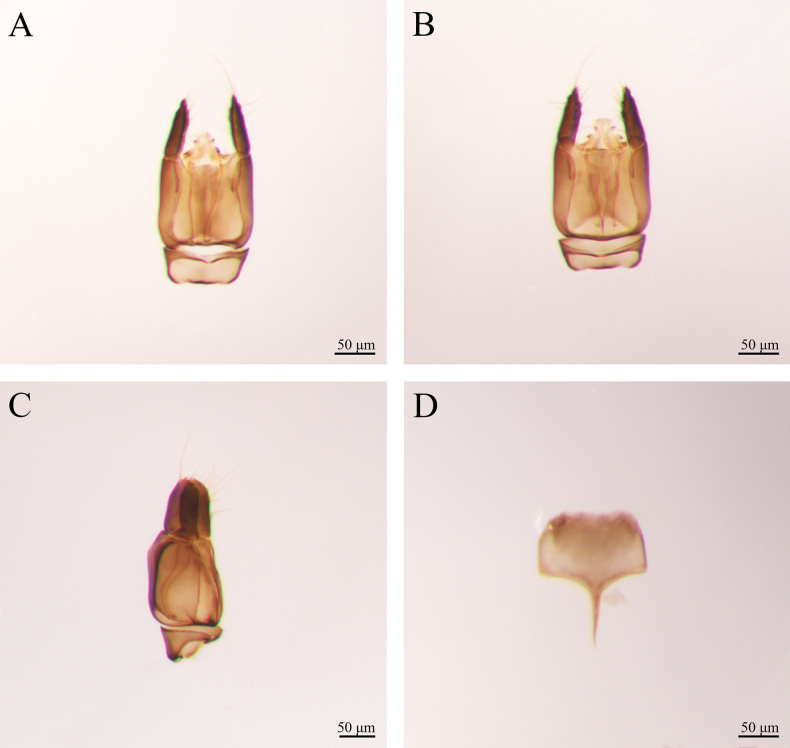
*Conostigmus clayulatus* Yang & Wang, sp. nov., male, holotype, genitalia. A. Dorsal view; B. Ventral view; C. Lateral view; D. S9.

***Male genitalia*** (Fig. 5). Harpe length slightly shorter than gonostipes, with numerous long and slender apical setae; harpe orientation: medial; harpe shape: simple and not bilobed, columnar; lateral setae of harpe present, but sparse. Parossiculus not fused. Gonostipes longer than wide, not fused with parossiculus. Penisvalva curved proximally. Gonossiculus and gonossiculus spine present; apical parossiculal setae present. Male S9 shape: stipitoplanar with a planar apex. The spiculum of S9 long, reaching 1/2 of S9. S9 has six setal patches at the end.

**Figure 6.**
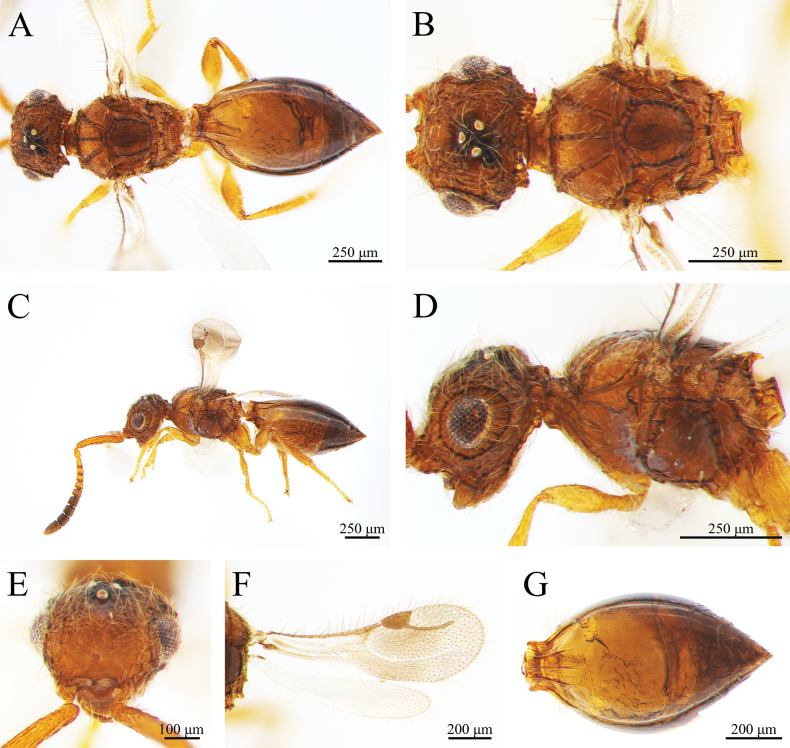
*Conostigmus clayulatus* Yang & Wang, sp. nov., female, holotype. A. Dorsal habitus; B. Head and mesosoma, dorsal view; C. Lateral habitus; D. Head and mesosoma, lateral view; E. Head, anterior view; F. Wings; G. Metasoma, dorsal view.

**Female** (Fig. 6). Same as the males, except for the following characters: body length = 1.7 mm; cranium, mesosoma and metasoma amber; scape, pedicel and F1–F5 aurantium, F6 to F9 brown; syntergum and metasoma gaster amber (Fig. 6G); antennal pedicel long, slightly longer than any individual flagellomere from F1 to F8 of the same individual; legs tawny, coxa amber. Wider mesosoma (length/width = 585/405 μm).

##### Remarks.

This new species is similar to *C. bipunctatus* Kieffer, 1907 in body length, axillular carinae, lateral propodeal carinae, but can be distinguished by the shorter, rod-shaped harpe (inverted U-shape in *C. bipunctatus*).

##### Distribution.

China (Chongqing).

##### Etymology.

Consistent with the genus name, the species name is a Latin masculine adjective meaning “clubbed”, referring to the small club-shaped harpe of the male genitalia in this species.

### ﻿Genetic distance and phylogenetic relationship

In this study, we obtained eight 28S rDNA sequences of five *Conostigmus* species. The genetic distances between *C. rotundus* Yang & Wang, sp. nov. and each of the other four *Conostigmus* species from China ranged from 0.003 to 0.020, and the genetic distances between *C. clayulatus* Yang & Wang, sp. nov. and each of the other four *Conostigmus* species from China ranged from 0.015 to 0.026 (Table 3). It confirms that these two species are new to the *Conostigmus*. Besides, *C. clayulatus* Yang & Wang, sp. nov. has pronounced sexual dimorphism. Female and male specimens of the two new species of *Conostigmus* both are genetically identical respectively.

**Table 3.** Genetic distance of 28S of ten *Conostigmus* and *Dendrocerus* species from China.

	1	2	3	4	5	6	7	8	9	10	11	12	13	14	15	16	17	18
1. *Dendrocerus carpenteri*, male																		
2. *Dendrocerus carpenteri*, female	0.000																	
3. *Dendrocerus laticeps*, male	0.007	0.007																
4. *Dendrocerus laticeps*, female	0.007	0.007	0.000															
5. *Dendrocerus bellus*, male	0.029	0.029	0.022	0.022														
6. *Dendrocerus bellus*, female	0.029	0.029	0.022	0.022	0.000													
7. *Dendrocerus anisodontus*, male	0.012	0.012	0.005	0.005	0.024	0.024												
8. *Dendrocerus anisodontus*, female	0.012	0.012	0.005	0.005	0.024	0.024	0.000											
9. *Dendrocerus lui*, male	0.029	0.029	0.022	0.022	0.041	0.041	0.020	0.020										
10. *Dendrocerus lui*, female	0.029	0.029	0.022	0.022	0.041	0.041	0.020	0.020	0.000									
11. *Conostigmus xui*, male	0.084	0.084	0.079	0.079	0.079	0.079	0.083	0.083	0.084	0.084								
12. *Conostigmus nankunensis*, male	0.095	0.095	0.092	0.092	0.088	0.088	0.092	0.092	0.094	0.094	0.019							
13. *Conostigmus ampullaceus*, male	0.086	0.086	0.081	0.081	0.081	0.081	0.084	0.084	0.086	0.086	0.002	0.017						
14. *Conostigmus ampullaceus*, female	0.086	0.086	0.081	0.081	0.081	0.081	0.084	0.084	0.086	0.086	0.002	0.017	0.000					
15. *Conostigmus rotundus*, male	0.090	0.090	0.084	0.084	0.084	0.084	0.088	0.088	0.090	0.090	0.005	0.020	0.003	0.003				
16. *Conostigmus rotundus*, female	0.090	0.090	0.084	0.084	0.084	0.084	0.088	0.088	0.090	0.090	0.005	0.020	0.003	0.003	0.000			
17. *Conostigmus clayulatus*, male	0.099	0.099	0.094	0.094	0.094	0.094	0.097	0.097	0.099	0.099	0.017	0.026	0.015	0.015	0.019	0.019		
18. *Conostigmus clayulatus*, female	0.099	0.099	0.094	0.094	0.094	0.094	0.097	0.097	0.099	0.099	0.017	0.026	0.015	0.015	0.019	0.019	0.000	

**Figure 7.**
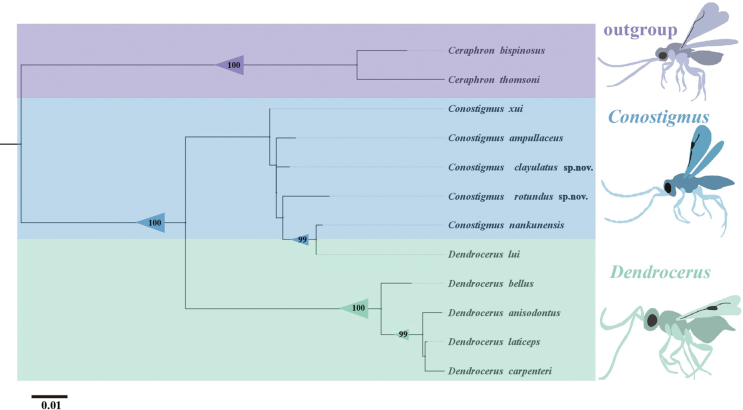
Relationships of *Conostigmus* and *Dendrocerus* based on maximum likelihood of 28S. Numbers refer to ultrafast bootstrap support values. The scale bar represents the number of substitutions per site.

We reconstructed a maximum-likelihood tree (ML tree) using 28S rDNA sequences from 12 species, including two *Ceraphron* species as outgroups, five species of *Conostigmus*, and five species of *Dendrocerus* as ingroups. The results of ML tree showed that *C. xui* diverged first, as the sister to the rest species. This clade was followed by *C. ampullaceus*. The topology was as (*C. xui* + (*C. ampullaceus* +(*C. clayulatus* + (*C. rotundus* + (*C. nankunensis* + *D. lui* Li & Wang, 2023))))) (Fig. 7).

## ﻿Discussion

The 28S rDNA sequences showed that the female and male of the same *Conostigmus* sepcies are genetically identical, which was consistent with the results of morphological identification, which implies that the 28S sequence can be used for matching male and female specimens of the same *Conostigmus* species.

Although the 28S sequences contained insufficient phylogenetic signal to reliably reconstruct overall relationships, they nonetheless support the assignment of *C. rotundus* Yang & Wang, sp. nov. and *C. clayulatus* Yang & Wang, sp. nov. to the genus *Conostigmus*.

## Supplementary Material

XML Treatment for Conostigmus

XML Treatment for Conostigmus rotundus

XML Treatment for Conostigmus clayulatus

## ﻿Additional information

### Use of AI

No use of AI was reported.

### Author ORCIDs

Yuanhan Yang https://orcid.org/0009-0009-5964-5835

Fang Li https://orcid.org/0009-0004-9269-7200

Yixin Huang https://orcid.org/0000-0002-7885-321X

Xu Wang https://orcid.org/0000-0001-6465-4052

Zhisheng Zhang https://orcid.org/0000-0002-9304-1789

Huayan Chen https://orcid.org/0000-0002-0382-1635

### Data availability

All of the data that support the findings of this study are available in the main text.
